# A dataset build using wearable inertial measurement and ECG sensors for activity recognition, fall detection and basic heart anomaly detection system

**DOI:** 10.1016/j.dib.2019.104717

**Published:** 2019-10-24

**Authors:** Adnan Nadeem, Amir Mehmood, Kashif Rizwan

**Affiliations:** a Faculty of Computer and Information System, Islamic University of Madinah, Madinah, Saudi Arabia; b Department of Computer Science, Federal Urdu University of Arts, Science & Technology, Pakistan; c Electronic Engineering Department, NED University of Engineering & Technology, Karachi, Pakistan

**Keywords:** Inertial sensors, ECG sensor, TUG test, Fall detection systems, ECG analysis, Daily life activities, SHIMMER^TM^

## Abstract

This paper defines two major data sets 1) from wearable inertial measurement sensors and 2) wearable ECG SHIMMER™ sensors. The first dataset is devised to benchmark techniques dealing with human behavior analysis based on multimodal inertial measurement wearable SHIMMER™ sensors unit during research studies “*Fall Detection System for the Elderly Based on the Classification of Shimmer Sensor Prototype Data*” [2] and “*A novel fall detection algorithm for elderly using SHIMMER wearable sensors*” [3]. The SHIMMER inertial sensor is a lightweight sensing device, incorporated with tri-axial accelerometer, a tri-axial gyroscope and tri-axial magnetometer, mounted on the waist of the subjects. The second dataset is developed to assess the feasibility of using SHIMMER™ wearable third generation ECG sensors for identification of basic heart anomalies by remote ECG analysis. The experimental protocol was carried out according to the Timed Up and Go (TUG) test [1], which is mainly used in fall detection and fall risk assessment systems specially designed for elderly. Three daily life activities such as standing still, walking and sitting on chair and getup were performed along with fall activity in controlled environment. This dataset is available on Data in Brief Dataverse [4] and a data repository [5].

**Table:** Specifications Table

Subject area	*Computer Science in healthcare*
More specific subject area	*Activity Recognition, Fall detection and basic anomaly detection in Heart functionalities*
Type of data	*Dimension of Data:* ***(NOTE: 14 attributes have been selected out of 26 attributes of Shimmer inertial sensor shown in sample file)*** *1. Provided data set contains 14 dimensional data collected form sensor, containing Time stamp raw, Time stamp in millisecond, acceleration raw (X axis), Acceleration cal (X axis), acceleration raw (Y axis), Acceleration cal (Y axis), acceleration raw (Z axis), Acceleration cal (Z axis), gyro raw (X axis), gyro cal (X axis), gyro raw (Y axis), gyro cal (Y axis), gyro raw (Z axis), and gyro cal (Z axis).* *Moreover,* *2. Activity set contains 3 kinds of activities i.e., L1: Standing still, L4: Walking, and L11: Stand to Sitting.* *And total number of records of each activity* *3. Eventually, 136 records of walk, 138 records of sit on chair and getup and 132 records of standing still activities have been observed.* *Hence, presented data set comprised of (136x14)* + *(138x14)* + *(138x14) equals to 5684 dimensions for inertial dataset* *Furthermore, ECG data set contains:* *1. 39 records* *2. 33 record contains 12 attributes: ECG Vx-RL calibrated, ECG Vx-RL raw, ECG LA-RA calibrated, ECG LA-RA raw, ExG2 CH1 calibrated, ExG2 CH1 raw, EXG1 Status raw, ECG LL-RA calibrated, ECG LL-RA raw, Timestamp raw, Timestamp calibrated, EXG2 Status raw* *3. 6 record contains 8 attributes: ECG LL-RA calibrated, ECG LL-RA raw, ECG LA-RA calibrated, ECG LA-RA raw, ECG Vx-RL raw, ECG Vx-RL calibrated, Timestamp raw, Timestamp calibrated* *Hence, presented ECG data set comprised of (33x12)* + *(6x8) equals to 444 main dimensions.* *ECG data set also contained a master data set file consisting 26 parameters regarding health condition of subjects with time and date are given.*
How data was acquired	*SHIMMER*^*TM*^*3*rd *generation inertial sensors were used for data collection from 114 subjects of different ages.*
Data format	*Timestamp, Raw and Calibrated data of Tri-Axial Accelerometer, Tri-Axial Gyroscope and ECG.*
Experimental factors	*Measure of dynamic behavior of subject's during different activities.*
Experimental features	*Data was incorporated with a total 136 records of walk, 138 records of sit on chair and getup and 132 records of standing still activities.*
Data source location	• *Department of Computer Science, University of Karachi, Pakistan.* • *Department of Computer Science, Federal Urdu University of Arts Science and Technology, Karachi, Pakistan* • *Dar ul Sukoon Elderly Home, Karachi, Pakistan* • *Edhi old age home, Pakistan* • *Islamic University of Madinah, KSA.*
Data accessibility	*Data is with this article and provided in the Data in Brief Dataverse*https://doi.org/10.7910/DVN/M7XKND *Also available at:*http://adnan-nadeem.com
Related research article	*Mehmood, Amir,* et al. *“A novel fall detection algorithm for elderly using SHIMMER wearable sensors.” Health and Technology (2019): 1–16.*https://doi.org/10.1007/s12553-019-00298-4

**Table:** 

**Value of the Data** • Inertial sensor dataset consists of three daily life activities based on timed up and go (TUG) test [1]. Collected by placing the inertial sensor on waist of the subjects, as it was considered as center of the body for minimal noise. • A fall event is included and that will be helpful to understand and compare with the behavior of an individual performing these activities in terms of acceleration and angling movements. • The data set can be used for developing fall detection systems especially for elderlies. • Also, this dataset included the ECG data which will be very helpful for anomaly detection in basic heart functionalities.

## 1 Data

In this data article, data coming from an inertial sensor device from SHIMMER™ [6] consisting a tri-axial accelerometer, triaxial gyroscope mounted on the waist of the subjects. A total of 114 subjects with different age and weight profiles were selected for performing voluntarily three activities of daily life routines as described in timed up and go (TUG) test [1], also included with a fall event in separate file. A written consent form was duly filled by selected subjects who were asked to perform activities from TUG test. This data may be used to develop fall detection systems specially for elderly population.

The collection of inertial sensors dataset was done using a C# application (discussed in next section) and generated in spreadsheets with naming pattern of shimmerXXX.xls where XXX were the numbers between 001 and 999 and each number represented the activities performed by each subject. Each record file contains attributes defined with examples in Table 1.

**Table 1:** An explained example of a data record from the dataset (inertial sensor).

Column	Meaning	Example
Time stamp raw	The time in raw format in which data sample was generated	640
Time stamp in millisecond	The time in millisecond in which data sample was generated	22019.53
Low Noise Accelerometer raw (X axis)	Acceleration at X axis of sensor device in raw format with low noise	2592
Low Noise Accelerometer calibrated (X axis)	Acceleration at X axis of sensor device in calibrated format (m/s^2^) with low noise	−0.04819
Low Noise Accelerometer raw (Y axis)	Acceleration at Y axis of sensor device in raw format with low noise	2051
Low Noise Accelerometer calibrated (Y axis)	Acceleration at Y axis of sensor device in calibrated format (m/s^2^) with low noise	−6.56627
Low Noise Accelerometer raw (Z axis)	Acceleration at Z axis of sensor device in raw format with low noise	1732
Low Noise Accelerometer calibrated (Z axis)	Acceleration at Z axis of sensor device in calibrated format (m/s^2^) with low noise	3.795181
Gyroscope raw (X axis)	Angular velocity at X axis of sensor device in raw format	−983
Gyroscope calibrated (X axis)	Angular velocity at X axis of sensor device in calibrated format (deg/s)	15.66412
Gyroscope raw (Y axis)	Angular velocity at Y axis of sensor device in raw format	−1026
Gyroscope calibrated (Y axis)	Angular velocity at Y axis of sensor device in calibrated format (deg/s)	15.00763
Gyroscope calibrated (Z axis)	Angular velocity at Z axis of sensor device in raw format	5304
Gyroscope calibrated (Z axis)	Angular velocity at Z axis of sensor device in calibrated format (deg/s)	−80.9771

**Fig. 1:**
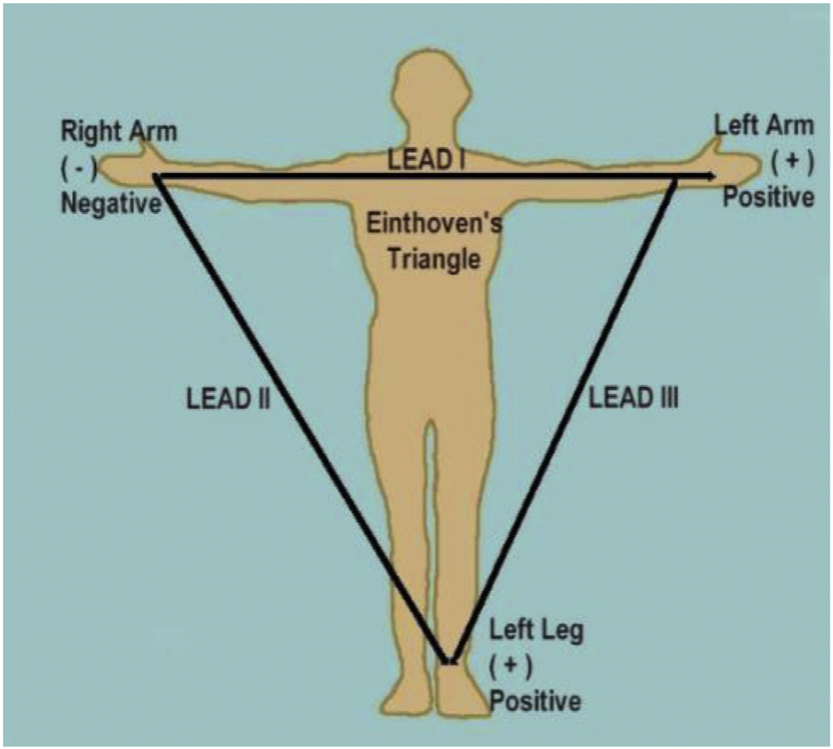
The Limb leads Configuration (Einthoven's triangle).

ECG data collection was collected using Android smartphone application, and data was saved in common separate vector (csv) file format. The ECG data acquired according to the Limb Lead I and limb Lead II configuration according to the Einthoven's triangle as shown in Fig. 1.

The column and row description were same as given in Table 1, but inertial sensors fields (accelerometer, gyroscope) were replace with Lead I (Left Arm and Right Arm) and Lead II (Right Arm and left Leg).

The Dataset was categorized by five age groups as aging is another critical factor that increases the probability of fall for a person as the age of a person increases, the chances of fall also increase. Hence the early fall detection in elderly may have a significant role in healthcare. The persons with age greater than 60 years are considered as elderly according to world health organization (WHO) report [7].

## 2 Experimental design, materials, and methods

**Fig. 2:**
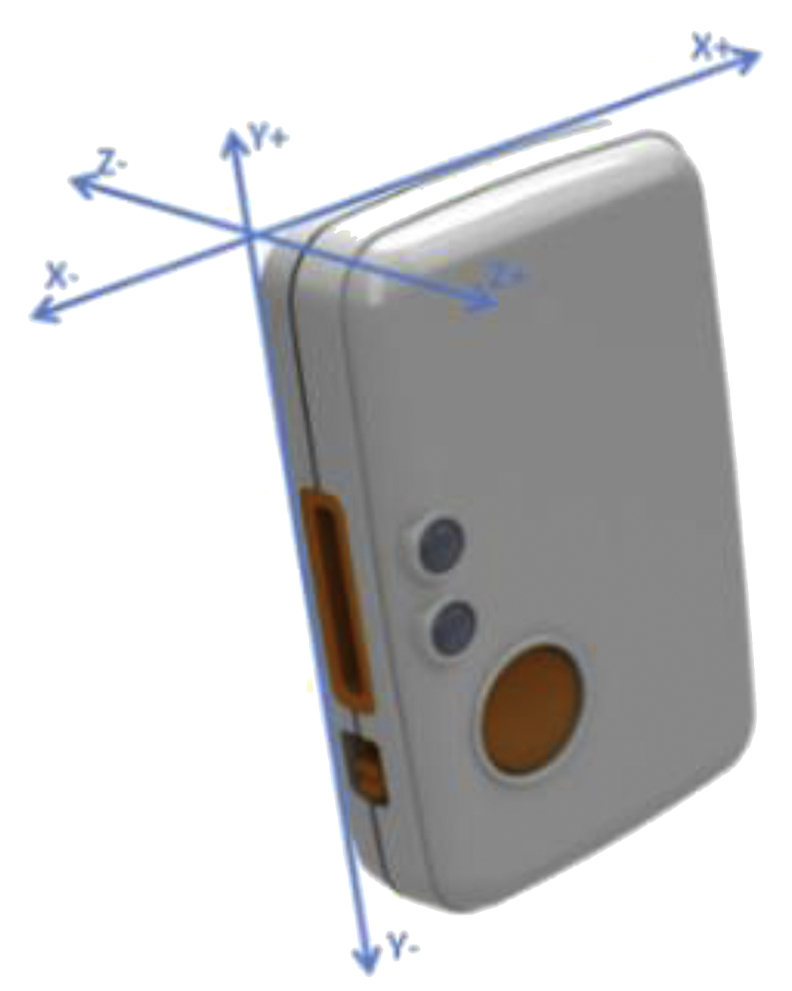
SHIMMER3 Sensor Device with default axis direction.

The Sensing Health with Intelligence, Modularity, Mobility and Experimental Reusability (SHIMMER) [6] mote is lightweight, and very tiny wearable sensor platform with Bluetooth class 2 connectivity used for data collection, allowing a flexible wireless support to various applications. It also enables the user to control over data capturing, for better interpretation. SHIMMER mote is incorporated with MSP430 microcontroller for processing and for communication it used ChipCon CC2420 radio with Revering Network RN-42 having a communication range up to 10 m, while default baud rate (transmission rate) is up to 115 K bauds. It also supports the external storage with maximum 2GB data storage in terms of micro SD card socket slot. The SHIMMER platform also have the support of software development for C#, MATLAB, Android and LabVIEW etc. Fig. 2 shows Shimmer3 inertial sensor device with three dimensions (x,y,z) allows user to wear with ease using a strap.

### 2.1 Inertial sensor data collection

**Fig. 3:**
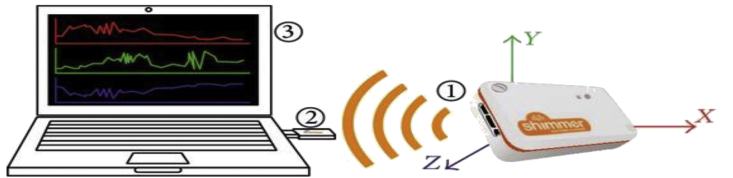
Data acquisition system.

Most studies such as [[8], [9], [10], [11]], suggest the optimal position of inertial sensor is waist, due to its nearness of center of the mass of the body, therefore, the readings of the waist mounted inertial sensors will not be affected and prevent the data from adding unwanted signal components (noise) by relational changes in the body movement of the subjects, enabling better recordings, also it is comfortable for the wearer [12]. The SHIMMER mote is connected through Bluetooth to a remote pc (Laptop in this case with Bluetooth dongle) as depicted in Fig. 3. where received data is stored for further processing through a logging application running on that laptop. The application contains multiple fields like user name, age, gender, height and weight. The sampling frequency was set to 51 Hz (51 data samples per seconds) which is adequate for acceleration data.

#### 2.1.1 Experiment scenario design

Before data collection, the height was measure using height scale adjusted on the wall, and weight was measured using weight machine of each subject. Subjects were asked to perform these activities in controlled environment. A line on the floor was marked on a distance of 5 m from the chair. The armless chair was used for sitting and getting up activity. Each activity was followed according to the defined in TUG test.

**Fig. 4:**
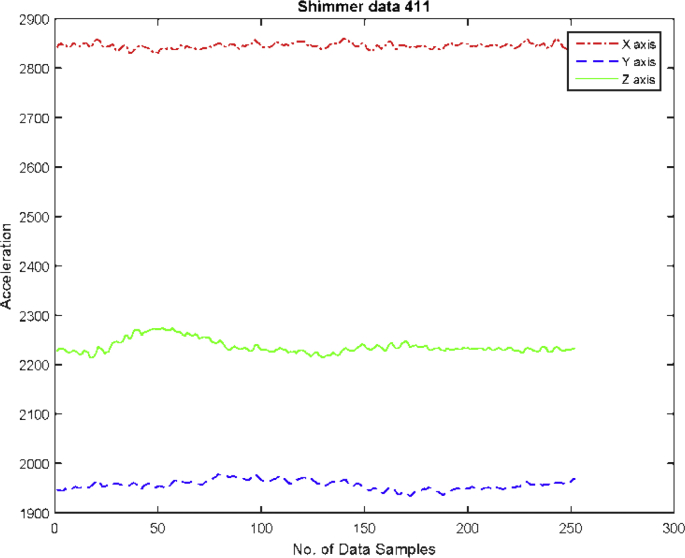
An example of standing position in graph (RAW data).
• ***Sitting on chair and getup from chair:*** This activity is an example of merged activities. The person first stands and then eventually sits as shown in graph of Fig. 5 as example of sit to stand to sit activity.

In Following, the experimental protocols for data collection are described for performing activities:

• ***Standing Posture:*** While collecting this posture, the subjects were asked to stand straight without any movement for 5 seconds. An example of data collection and raw data (in graph form) of standing position shown in Fig. 4.

**Fig. 5:**
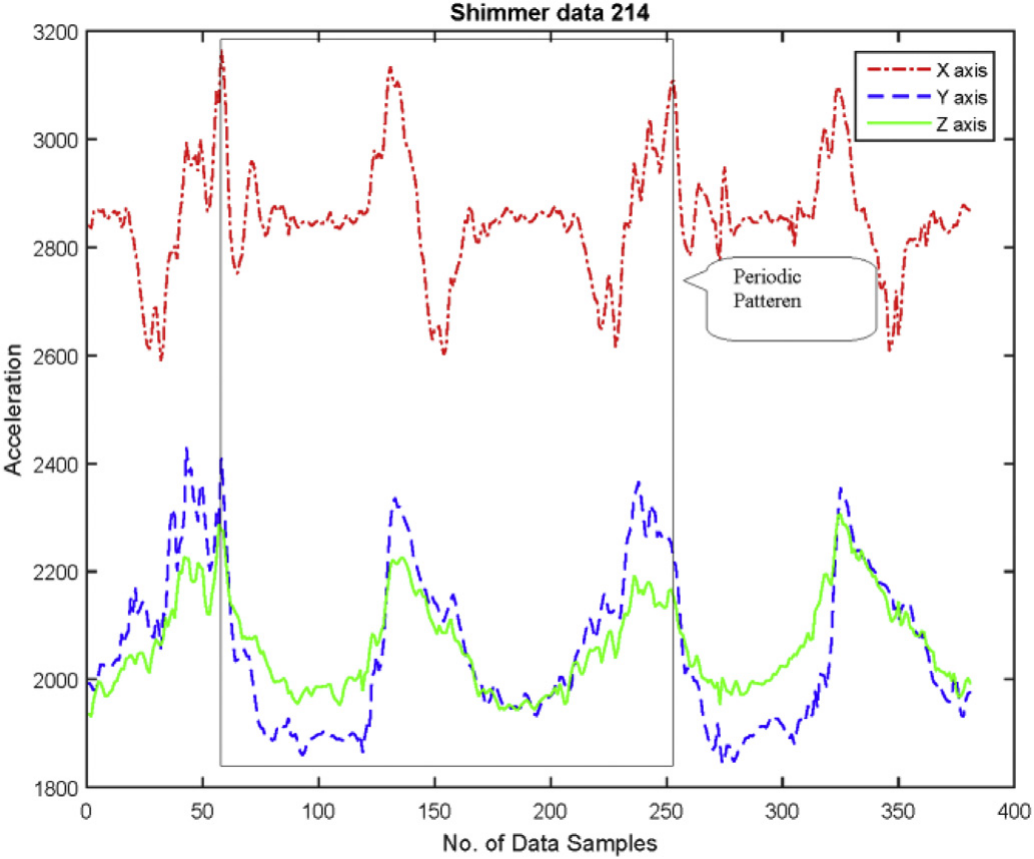
An example of sit to chair and get up in graph (RAW data).
• ***Walking:*** For this activity, subjects were asked to walk on a leveled surface for approximately 5 m, while the graph of walking pattern can be seen in Fig. 6.

**Fig. 6:**
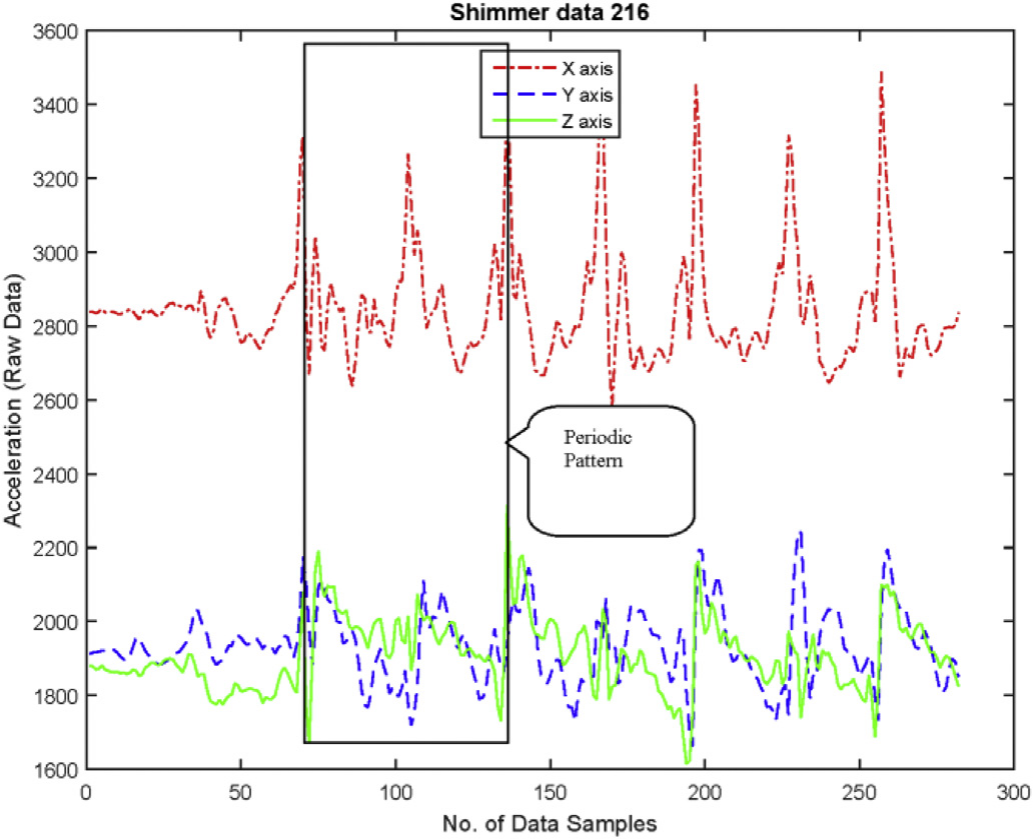
An example of walk activity in graph (RAW data).
• ***Fall:*** Four subjects were asked to perform fall event intentionally on a mattress. Since this is not an unintentional fall, but it shows a notable change from other collected ADLs as depicted in Fig. 7.

**Fig. 7:**
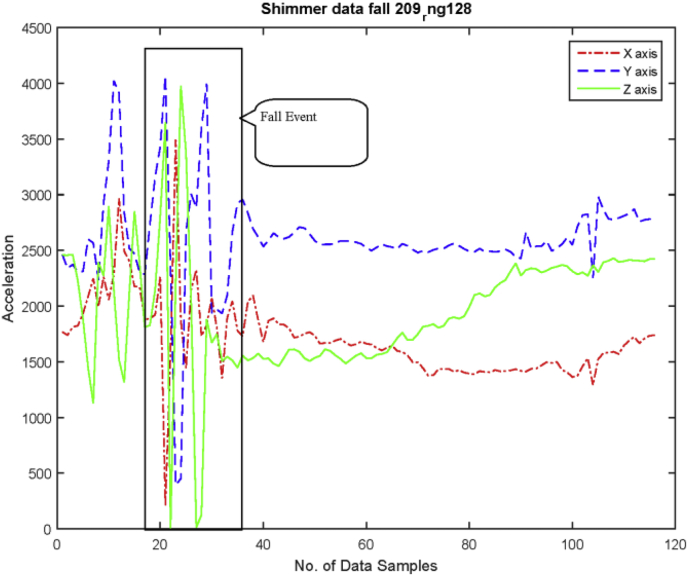
An example of intentionally fall in graph (RAW data).

Each and every activity has a unique pattern. It may very person-to-person but not that much. Therefore, for basic fall detection and behavioral analysis, this data set may be helpful. We will increase the number of fall and data set size in near future.

### 2.2 ECG data collection

**Fig. 8:**
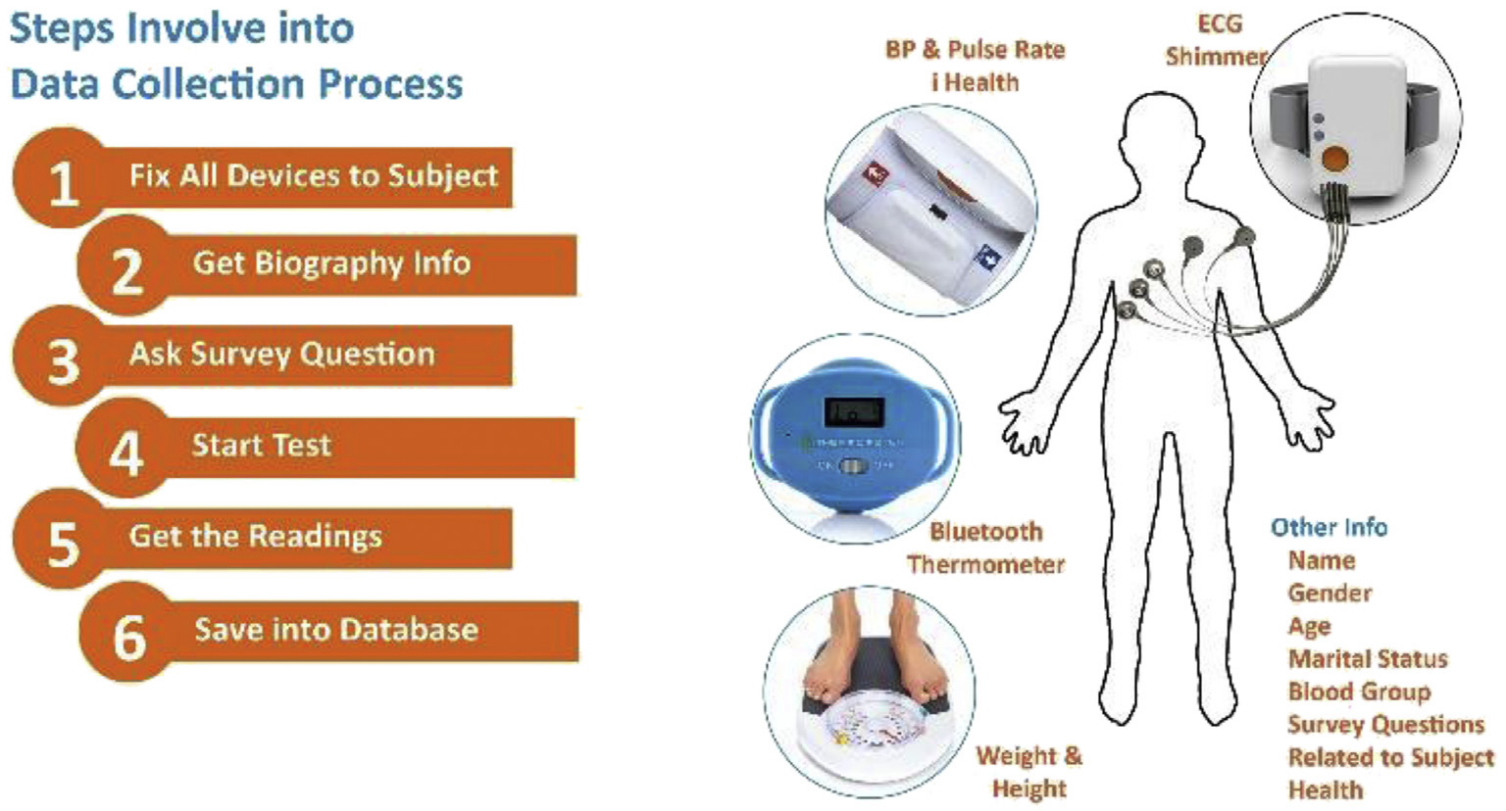
Steps involve in ECG data collection.

The ECG data collection was divided in six different steps which took approximately 10–15 minutes to complete the whole procedure. For corrected and proper ECG recordings, Blood Pressure and pulse rate from digital Bluetooth device (i-health), a digital Bluetooth thermometer and a weight machine were used to identify the body parameters of the subjects as shown in Fig. 8.

#### 2.2.1 Experiment scenario design

An Android smartphone application was developed for recording the basic information of the subjects such as age, weight, history of heart disease, gender, married or unmarried etc. as shown in Fig. 8. The steps involved in ECG data collection are follows:

• Take Biography info using mobile application.
• Take weight, height using weight machine & height scale.
• Get body temperature using sensing thermometer.
• Fix “iHealth” device to the subject.
• Fix ECG 5 Electrodes leads to the subject chest and connect with SHIMMER Sensor.
• Start test that will take reading from both devices.
• After the procedure electrodes are removed and discharge.
• Electrodes paste is wiped off with a damp cloth.

The study involves data collection of certain daily life activities through wearable SHIMMER sensor on normal human subjects during research studies [[2], [3]]. All procedures followed were in accordance with the ethical standards of the Helsinki Declaration of 1975, as revised in 2008. Informed consent was obtained from all patients for being included in the study. This dataset is available on Data in Brief Dataverse [4] and a data repository [5].
